# CsrB, a noncoding regulatory RNA, is required for BarA-dependent expression of biocontrol traits in *Rahnella aquatilis* HX2

**DOI:** 10.1371/journal.pone.0187492

**Published:** 2017-11-01

**Authors:** Li Mei, Sanger Xu, Peng Lu, Haiping Lin, Yanbin Guo, Yongjun Wang

**Affiliations:** 1 College of Forestry and Biotechnology, Zhejiang A&F University, Lin’An, China; 2 Department of Ecological Science and Engineering, College of Resources and Environmental Sciences, China Agricultural University, Beijing, China; 3 National and Provincial Joint Engineering Laboratory of Bio-pesticide Preparation, Lin’An, China; Meharry Medical College, UNITED STATES

## Abstract

**Background:**

*Rahnella aquatilis* is ubiquitous and its certain strains have the applicative potent as a plant growth-promoting rhizobacteria. *R*. *aquatilis* HX2 is a biocontrol agent to produce antibacterial substance (ABS) and showed efficient biocontrol against crown gall caused by *Agrobacterium vitis* on sunflower and grapevine plants. The regulatory network of the ABS production and biocontrol activity is still limited known.

**Methodology/Principal findings:**

In this study, a transposon-mediated mutagenesis strategy was used to investigate the regulators that involved in the biocontrol activity of *R*. *aquatilis* HX2. A 366-nt noncoding RNA CsrB was identified *in vitro* and *in vivo*, which regulated ABS production and biocontrol activity against crown gall on sunflower plants, respectively. The predicted product of noncoding RNA CsrB contains 14 stem-loop structures and an additional ρ-independent terminator harpin, with 23 characteristic GGA motifs in the loops and other unpaired regions. CsrB is required for ABS production and biocontrol activity in the biocontrol regulation by a two-component regulatory system BarA/UvrY in *R*. *aquatilis* HX2.

**Conclusion/Significance:**

The noncoding RNA CsrB regulates BarA-dependent ABS production and biocontrol activity in *R*. *aquatilis* HX2. To the best of our knowledge, this is the first report of noncoding RNA as a regulator for biocontrol function in *R*. *aquatilis*.

## Introduction

All organisms contain a wealth of noncoding regulatory RNAs (ncRNAs) that function in a variety of cellular processes [1]. The ncRNAs play key roles in many physiological and adaptive responses in bacteria. The ncRNAs act at the post-transcriptional level and utilize less energy and time for synthesis and turnover [2]. It is more advantageous that bacteria use ncRNA-mediated responses than the regulation by protein transcriptional factors in the rapidly changing environments [3]. Most of the known ncRNAs are divided into two categories: *cis*-encoded ncRNAs that are transcribed in the antisense orientation to their target protein-encoding mRNA, and *trans*-encoded ncRNAs that are transcribed from intergenic regions with multiple targets [3]. Numerous ncRNAs have been described in *Escherichia coli* and were estimated to several hundred on the basis of genome sequences [4].

In *E*. *coli*, ncRNAs (e.g., CsrB and CsrC) have a high affinity for certain RNA-binding proteins that act as translational repressors. For example, CsrA, a carbon storage regulator, is a recently discovered as a global regulatory system that controls bacterial gene expression on the post-transcriptional level [5, 6]. Repeated ANGGA motifs (N is any nucleotide) in target mRNAs are binding sites for CsrA [5]. Translation of the mRNA was repressed when the most distal of such motifs coincided with the ribosome-binding sites [7]. The noncoding regulatory Csr RNAs have flower-like secondary structures with multiple GGA motifs in unpaired regions. The regulatory ncRNAs containing multiple CsrA binding sites within the loops of predicted stem-loops competed with mRNAs for CsrA binding, thus antagonized CsrA activity [8]. Homologs of CsrA (e.g. RsmA in *Pseudomonas aeruginosa*) were highly conserved and identified in diverse bacteria to play key roles in biofilm formation and dispersal [9] as well as in regulating virulence factors of animal and plant pathogens [10–14]. In the proteobacteria, most of the bacterial species having CsrA homologs also contained the homologs of BarA and/or UvrY (e.g. the GacA–GacS two component system in *P*. *aeruginosa*) and their interaction network among these proteins has been studied in several bacterium species [3]. Besides these pathogenic bacteria, ncRNAs have also been demonstrated in the certain plant-beneficial soil bacteria. Three ncRNAs including RsmY, RsmZ, and RsmX were key factors to relieve RsmA-mediated regulation of secondary metabolism and biocontrol traits in the GacS/GacA cascade of *P*. *fluorescens* CHA0 [15–17]. The ncRNAs mutants in *P*. *fluorescens* CHA0 were strongly impaired in its biocontrol properties in a cucumber-*Pythium ultimum* microcosm [15].

The gram-negative bacterium *Rahnella aquatilis* is ubiquitous and its certain strains have the applicative potents as plant growth-promoting rhizobacteria. The functions of *R*. *aquatilis*, including fixing nitrogen, solubilize mineral phosphate, and biocontrol activity were described previously [18–22]. The nonpathogenic strain *R*. *aquatilis* HX2 was isolated from vineyard soils and showed to suppress grapevine crown gall disease caused by *Agrobacterium vitis* [19, 20]. The biocontrol ability of strain HX2 was related to the production of an antibacterial substance (ABS) and exhibited a broad spectrum of inhibition activity against phytopathogenic bacteria for ABS production regulation by pyrroloquinoline quinone (PQQ) and the BarA/UvrY two component regulatory system [23–25]. Here, a transposon-mediated mutagenesis strategy was used to investigate the factors that regulated the biocontrol activity of *R*. *aquatilis* HX2. A novel regulatory ncRNA was identified, located and characterized. The relevance of this regulatory ncRNA to growth and beneficial activities of strain HX2 are assessed with respect to antibacterial activity and biocontrol activity of sunflower crown gall disease.

## Materials and methods

### Bacterial strains, plasmids, and culture conditions

The bacterial strains and plasmids used in this study are listed in Table 1. *R*. *aquatilis* strains were cultured at 28°C on potato dextrose agar (PDA) medium or with agitation (170 rpm) in potato-dextrose broth (PDB) [20]. *E*. *coli* strains were grown at 37°C on Luria-Bertani (LB) medium. *Agrobacterium vitis* strain K308 was grown either on yeast extract broth YEB or YEB agar at 28°C [26]. When required, the growth of *R*. *aqautilis* and *E*. *coli* were performed in media with the filter-sterilized antibiotics (50 μg ml^-1^ kanamycin; 50 μg ml^-1^ ampicillin; 20 μg ml^-1^ tetracycline; 5 μg ml^-1^ gentamicin).

**Table 1:** Bacterial strains, plasmids, and DNA primers used in this study.

Strains, plasmids and primers	Character ^a^	Sources or references
**Strains**		
***Escerichia coli***		
DH5α	F- Δ(*lac*-*argF*)*U169 recA-1 endA-1 hsdR* (r^-^_K_ m^-^_K_) *supE-44 gyrA-1 relA-1* deoR *thi-1* (Φ80d*lac-Z* ΔM15)	This laboratory
DH5α (λ-pir)	*thi pro hsdR hsdM*^+^ *recA* RP4-2 Tc::Mu-Km::Tn7 λ-pir	This laboratory
***Rahnella aquatilis***		
HX2	wild type, ABS^+^, Ap^R^	This laboratory
MR57	HX2 derivative with Δ*barA* mutant, Ap^R^	[25]
MR57csrB	MR57 containing pRK*csrB*, Ap^R^ Km^R^	This study
MR57barA	MR57 containing pRK*barA*, Ap^R^ Km^R^	[25]
TR61	HX2 derivative containing a Tn5 insertation in *csrB* loci, Ap^R^ Km^R^,	This study
MR61	HX2 derivative with ~200 bp deletion in *csrB*, Ap^R^	This study
MR61csrB	MR61 containing pRK*csrB*, Ap^R^, Tc^R^,	This study
MR61barA	MR61 containing pRK*barA*, Ap^R^, Tc^R^,	This study
***Agrobacterium vitis***		
K308	Pathogen of grapevine and sunflower crown gall	This laboratory
**Plasmids**		
pBS	pBluescript II SK+, ColE 1, cloning vector, Ap^R^;	Stratagene
pUTkm1	Delivery plasmid for Tn5, R6K replicon, Ap^R^ Km^R^	[29]
pML122	RSF1010-derived expression and *lac*-fusion broad host-range vector, Gm^R^	[37]
pRK600	ColE1, oriV, RP4; *tra*+;RP4 oriT, helper plasmid in triparental matings, Cm^R^	[33]
pSR47S	*sacB*, oriT, Km^R^,	[32]
pRK415G	Broad-host-rang cloning vector, IncP1 replicon, Gm^R^ Tc^R^	This laboratory
pSRΔcsrB	pSR47S containing a ~2000 bp *Kpn* I-*Xba* I fragment with *csrB* deletion Km^R^	This study
pRKcsrB	pRK415G containing 1300 bp *Hind* III / *Kpn* I fragment including *csrB*	This study
pRKbarA	pRK415G containing *barA*	[25]
pMLcsrBlac	pML122 containing transcriptional *csrB*-*lacZ* fusion	This study

^a^ Ap^R^, Cm^R^, Km^R^, Gm^r^, and Tc^R^ indicate resistance to ampicillin, chloromycetin, kanamycin, gentamicin, and tetracycline, respectively.

### General genetic manipulation

Isolation of genomic DNA from strain HX2 and plasmid DNA from *E*. *coli* was performed according to standard procedures [27]. Restriction enzyme digestions were carried out as recommended by the manufacture (TaKaRa, Japan) while ligations were applied T4 DNA ligase (TaKaRa). Ex Taq DNA polymerase (TaKaRa, Japan) was used for PCR amplification of inserts. DNA sequencing was performed (Invitrogen Life Technologies, Beijing, China) and analyzed by using BLAST server of the National Center for Biotechnology Information (http://www.ncbi.nlm.nih.gov/BLAST). Primers was designed according to the whole genome sequence of *R*. *aquatilis* HX2 (accession number CP003403-6) [28] and listed in Supporting Information file (S1 Table).

### Transposon mutagenesis and screening of ABS-deficient mutants

Tn5 mutagenesis of strain HX2 was accomplished by conjugal mating with *E*. *coli* S17-1(pUTkm1) [29]. Transconjugants grown within 48 to 60 h were assayed for inhibiting growth of *A*. *vitis* strain K308 *in vitro* as previously described [19]. The mini-Tn5-disrupted genes in these transconjugants were identified by cloning and sequencing the genomic DNA fragments flanking the transposon.

### Determination of CsrB 5’-end

The 5’-end of *csrB* transcript was mapped by rapid amplification of its cDNA end (RACE) [30]. Briefly, total RNA from strain HX2 was extracted from cells collected at late stationary phase. Reverse transcription (RT) reaction was performed using First-Strand cDNA Synthesis kit (TaKaRa) according to the instruction. T4 RNA ligase (Promaga was used to anchor a 5’-phosphorylated, 3’-end ddATP-blocked oligonucleotide DT88 (P-DT88-ddATP) to the single-stranded cDNA. The resulting ligation mixture was used in the subsequent semi-nested PCRs. The anchor-ligated cDNA was amplified with primers DT89 (anchor-specific primer) and csrBRA1, followed by amplification with primer DT89 and the internal primer TRR11. A 228-bp amplicon was purified and blunt-end cloned into the plasmid pBS (Table 1). Three independent clones were selected and sequenced.

### Construction of *csrB* in-frame deletion mutant

In-frame deletions of *csrB* were constructed utilizing a two-step homologous recombination strategy [31]. Primers were designed based on the upstream and downstream of *csrB* sequences for PCR fragment amplification from the genome of HX2 (Table 1). Briefly, primers csrBKO1 and csrBKO2 were used to amplify a 1042-bp region upstream of *csrB* while another 1104-bp fragment created by primers csrBKO3 and csrBKO4 is the downstream of *csrB*. The primer csrBKO2 shares the identical 22-bp oligonucleotides with the primer csrBKO3. A PCR-after-ligation was performed after mixture with these above amplified DNA fragments using the primer csrBKO1and csrBKO4 for generation of a 214-bp deletion site within 2124-bp *csrB* fragment. PCR reactions was initiated at 15 min at 94°C, followed by 35 cycles of 45 sec at 94°C, 40 sec at 66°C and 1 min at 72°C, and performed final extension at 72°C for 10 min. After being digested with *Not* I, the DNA fragment was ligated into pSR47S to obtain pSRΔcsrB [32]. This suicide plasmid was transformed into the *E*. *coli* DH5α (λ-pir) and mobilized from DH5α (λ-pir) into the wild-type *R*. *aquatilis* strain HX2 by triparental mating with helper plasmid pRK600 [33]. Exoconjugates were selected on ABM agar plates containing kanamycin and second recombination events were chose according to the previous methods [23].

### Genetic complementation of the CsrB mutant

To complement the CsrB mutant, a 1300-bp csrBCO1 and csrBCO2 amplified DNA fragment containing the *csrB* was cloned into the broad host range vector pRK415G resulting in the complementation plasmid pRKcsrB (Table 1). The plasmid pRKcsrB was mobilized into the MR57 or MR61 strain by tri-parental mating for complementation.

### Construction of a *csrB*-*lacZ* fusion

A reporter plasmid was constructed carrying a transcriptional *csrB*-*lacZ* fusion in which the +1 nucleotide of *lacZ* corresponds to the +1 nucleotide of the *csrB* promoter. The *csrB* promoter was PCR-amplified with primers csrBPR1 and csrBPR2. PCR products were digested with *Bam*H I and *Pst* I and ligated to pML122 to produce the construct pMLcsrBlac that was confirmed by sequencing.

### Antibiosis test *in vitro* and biocontrol crown gall disease in greenhouse

The antagonist *R*. *aquatilis* HX2 and its derivative strains were tested for antibiosis *in vitro* against pathogenic strain *A*. *vitis* K308 via a modified Stonier′s method [19]. Biocontrol activity assays were performed on sunflower (*Helianthus annuus* L. cv. Frankasol) stems with two true leaves grown in a greenhouse. Briefly, the suspension of pathogenic bacterial strain K308 (ca. 2 ×10^8^ CFU ml^-1^) was mixed with an equal volume of HX2 and/or its derivative strains suspension (ca. 2 × 10^8^ CFU ml^-1^). A 10-μl suspension drop of bacterial mixture was injected into a 1.0 cm longitudinal incision in sunflower stem. The inoculation site was wrapped with Parafilm and incubated for 15 days post-inoculation (dpi). Thereafter, galls were formed and excised to weigh biomass. Sterile buffered saline (SBS, 0.85% NaCl) was applied as a negative control while K308 mixed with SBS was served as a positive control. The effectiveness index (EI) was calculated using the following formula: EI (%) = [(*C*-*T*)/*C*]×100, where *C* is the mean fresh weight of the crown gall tumor of the positive control group and *T* is the mean fresh weight of the crown gall tumor in the treated group. The assay was independently performed with four replicates containing 10 plants per treatment.

### qPCR analysis

Bacterial strains were grown in PDB and total RNA was isolated by using the TRI reagent method and Turbo DNA-free DNase kits (TaKaRa) as suggested. cDNA was synthesized with 0.5 μg of total RNA as template. qPCR was performed to quantify the transcriptional level of target genes in different samples using RT Master Mix (TaKaRa) and the qTOWER 2.0/2.2 Real Time PCR System (Analytik Jena, Germany) following the protocols of manufactures. The *rpl*U was used as the endogenous control for data analysis. Data were analyzed using Relative Expression Software Tool [34].

### Statistical analysis

Data were analyzed using ANOVA program of the SAS software (version 8.2; SAS, Inc., Cary, NC). Mean with stand error was tested with a Student’s *t*-test (*P*< 0.05).

## Results

### Isolation and characterization of an ABS-deficient mutants in *R*. *aquatilis* HX2

A total number of 2140 mini Tn5-induced mutants of *R*. *aquatilis* HX2 with kanamycin resistance were assayed for ABS production. A mutant TR61 that significantly reduced the inhibition of *R*. *aquatilis* HX2 against *A*. *vitis* K308 on PDA plates was obtained (Table 2). A single Tn5 insertion was present in TR61 by Southern blotting and further indicated by a single hybridizing band upon digestion with *Kpn* I, *Sal* I, or *Pst* I (data not shown). Physiological and biochemical characteristics between HX2 and TR61 were partially not different [35]. All data showed consistent to wild-type strain HX2 for catalase, citrate utilization, Voges-Proskauer, and D-glucose oxidation, and they exhibited negative reaction for oxidase or methyl red (Table 2). Particularly, the mutant TR61, exhibited to reduce biological control ability to grapevine crown gall with 14.2 of EI of the strain TR61 compared to 96.3 of EI of wild type strain HX2.

**Table 2:** Inhibition effects of *Rahnella aquatilis* HX2 and its derivative strains on growth of *Agrobacterium vitis* strain K308 and tumor formation on sunflower (*Helianthus annuus* L. cv. Frankasol) seedlings.

Strain	Inhibition zone diameter (mm)^a^	EI(%)^b^
HX2	25.3 a	96.3 a
MR57	5.2 b	10.2 b
MR57(pRKcsrB)	26.1 a	/
MR57(pRKbarA)	24.5 a	/
TR61	5.1 b	14.2 b
MR61	4.6 b	12.5 b
MR61(pRKcsrB)	25.8 a	/
MR61(pRKbarA)	5.1 b	/

^a^ HX2 and its derivative strains were spot inoculated onto PDA medium and incubated at 28°C for 24 h. Production of ABS was assessed by overlaying the plates with a suspension of *A*. *vitis* K308 as the indicator, as described previously [20]. Data with the same letters in the same column are not significantly different (*P*<0.05).

^b^ EI was calculated by the formula EI (%) = [(*C*-*T*)/*C*]×100, where *C* is the average fresh weight of the crown gall tumor of the control group (*A*. *vitis* K308 only) and *T* is the average weight of the crown gall tumor of the treated group. Galls were excised and weighed 42 days after inoculation. Data are means of three replicates. Data with the same letters in the same column are not significantly different (*P*<0.05).

### Charaterization of a small noncoding RNA CsrB in *R*. *aquatilis*

**Fig 1:**
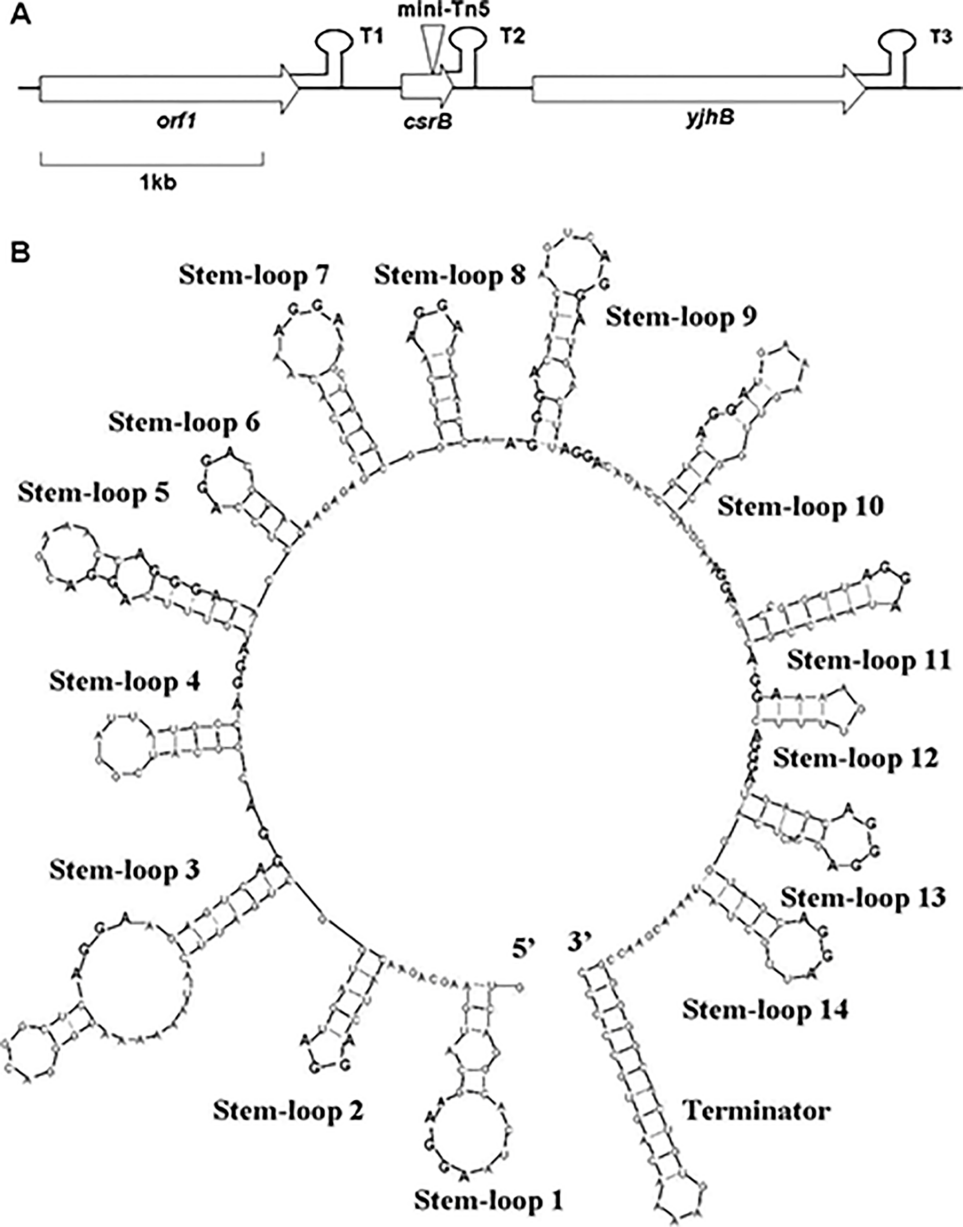
Characteristics of CsrB of *Rahnella aquatilis* HX2. (A) The region of *R*. *aquatilis* HX2 with *csrB*, *yjhB* and an unknown gene. T1, T2 and T3 are ρ-independent terminators. The mini-Tn5 inserted site in *R*. *aquatilis* TR57 was pointed by the triangle. (B) Predicted secondary structure of CsrB at 28°C using the MFOLD program (Zuker et al., 1999; http://www.bioinfo.rpi.edu/applications/mfold/old/rna/form1-2.3.cgi). AGGA and AGGGA motifs are in bold.

Genomic DNA was digested with *Hin*d III and ligated for transformation by *E*. *coli* DH5a. The resulting plasmids of *E*. *coli* colonies with kanamycin resistance were screened by PCR. The flanking sequence of Tn5 transposon in TR61 mutant resulted in an unknown locus (Fig 1A). This locus did not appear to contain any open reading frames and has a potential ρ-independent terminator (Fig 1B). Transcriptional start site of this locus was mapped and identified by RACE (Data not shown). Analysis using Ribosome Binding site calculator v 2.0 [36] indicates that this locus is unlikely to be translated because typical rates of translation were absent using all possible start codons. A model of computing-simulated folding [37] of this 366-nt locus transcript at 28°C was predicted 14 stem-loop structures and another ρ-independent terminator harpin with 23 characteristic GGA motifs in the loops and other unpaired regions. Modeled secondary structure was resembled like the noncoding regulatory C*srB* in *E*. *coli*. Therefore, *csrB* was referred to this locus in *R*. *aquatilis* HX2.

### CsrB is associated with ABS production and biocontrol in *R*. *aquatilis*

**Fig 2:**
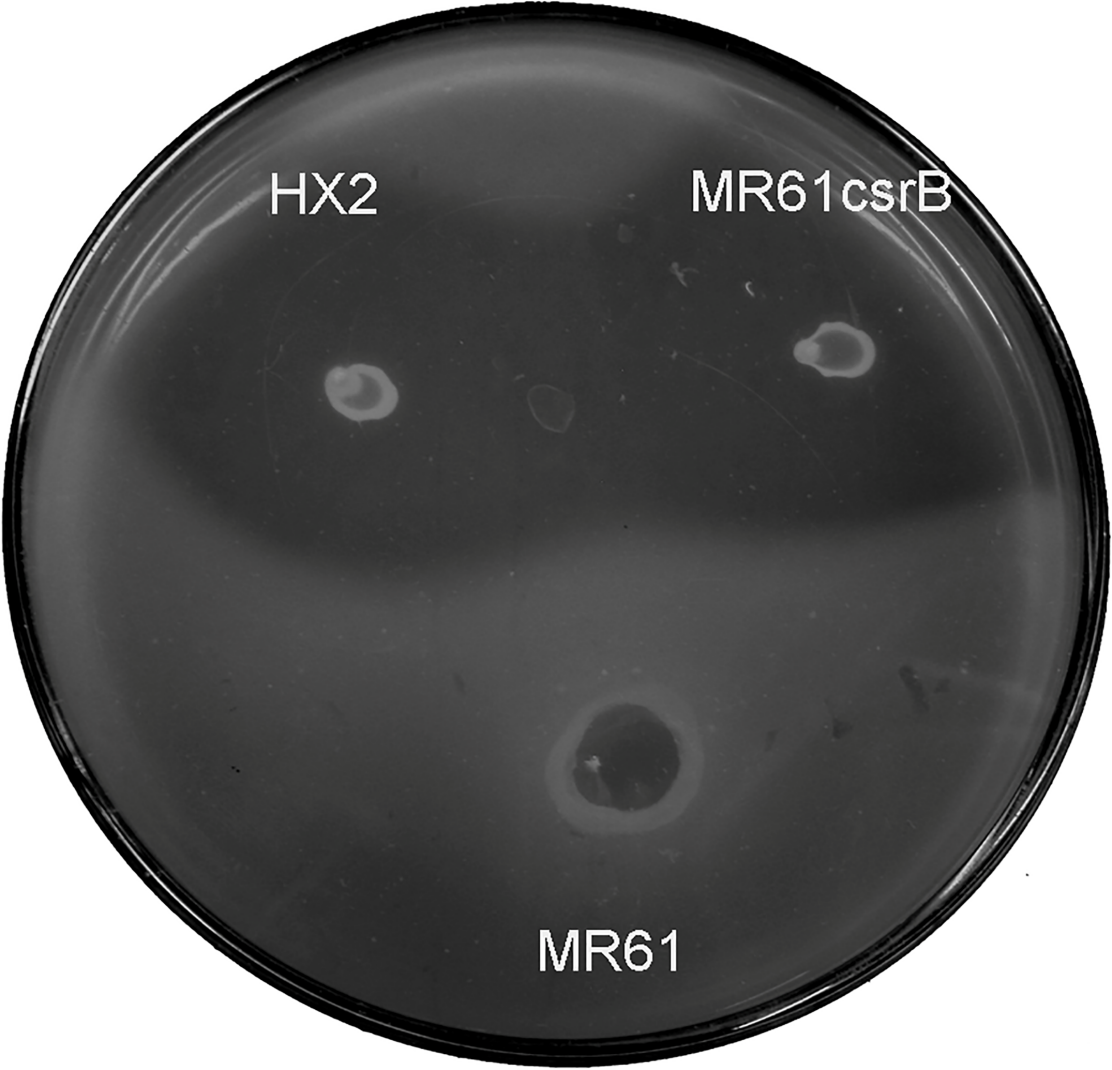
Inhibition zone by *Rahnella aquatilis* HX2, and its derivatives (*R*. *aquatilis* TR61 and *R*. *aquatilis* MR61) against *Agrobacterium vitis* K308.

**Fig 3:**
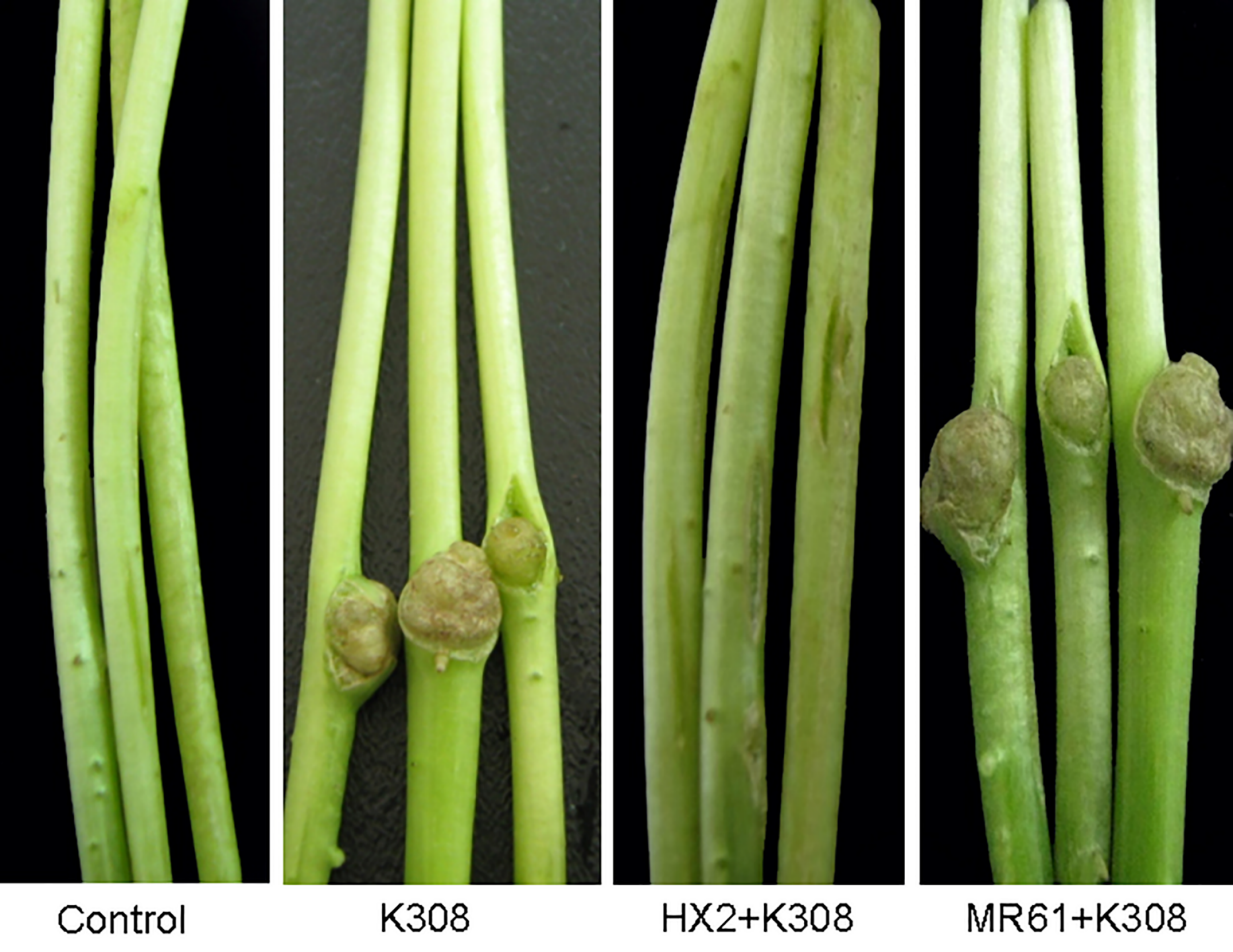
Biological control phenotypes of wild-type *Rahnella aquatilis* HX2 and its derivative strains on sunflower (*Helianthus annuus*) shoots. Shoots were inoculated by placing 10-μl drops of bacterial strains (10^8^ CFU ml^-1^), alone or in equal-number mixtures, at sites in which wounds of 1.0-cm longitudinal incisions were made with a sterile scalpel. Tumor were readily apparent 7 weeks after inoculation. The treatments were SDW alone (Control), *Agrobacterium vitis* K308 alone (K308), *R*. *aquatilis* HX2 plus *A*. *vitis* K308 (HX2+K308), and *R*. *aquatilis* MR61 plus A. vitis K308 (MR61+K308).

This locus referring to the CsrB RNA was disrupted by the homologous recombination and generated mutant strain MR61. The mutant was verified by PCR and DNA sequencing by the outside flanking region from the according mutants (data not shown). Mutant MR61 exhibited reduced the ability of inhibiting growth of *A*. *vitis* K308 (Fig 2 and Table 2). Strain MR61csrB, which is the complemented strain generated by plasmid pRKcsrB containing the *csrB*, displayed the similar or same phenotypes of *A*. *vitis* inhibition as wild type strain HX2 (Fig 2 and Table 2). To determine the function of the ncRNA CsrB in the biocontrol traits of *R*. *aquatilis* HX2, the biocontrol ability of strains were performed on the sunflower plants against gall disease caused by *A*. *vitis* K308. Strain HX2 caused to less infection in sunflower plants. However, the CsrB mutant strain MR61 exhibited deficient biocontrol activity to crown gall disease on sunflower plants with 12.5 and 96.3 of EI in MR61 and HX2, respectively (Fig 3, Table 2).

### CsrB is regulated by BarA in *R*. *aquatilis* HX2

**Fig 4:**
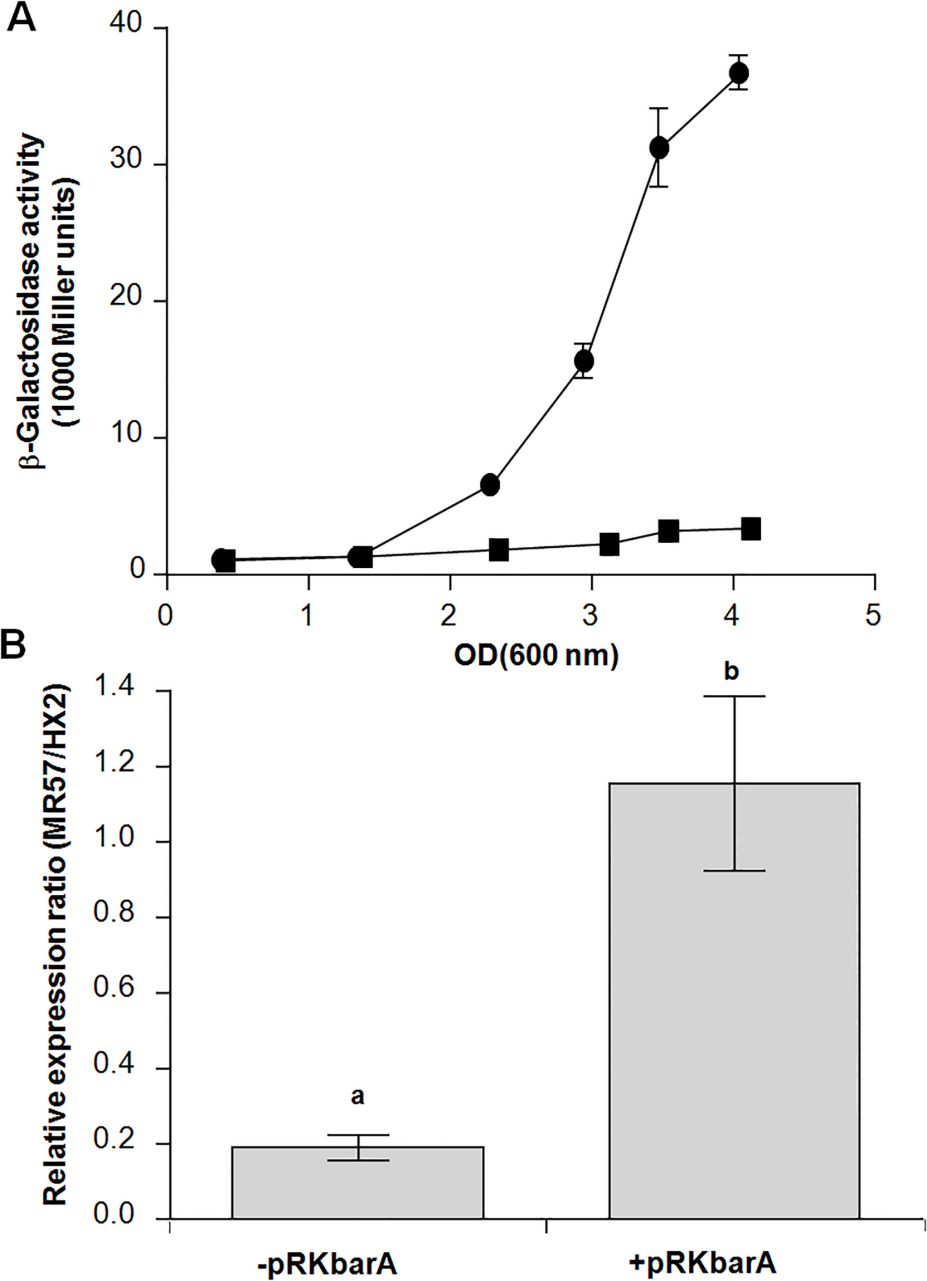
The ncRNA CsrB is controlled by BarA at the posttranscriptional level in *Rahnella aquatilis* HX2. (A) Activation of *csrB* transcription by BarA. β-galactosidase activities of a transcriptional *rsmB*-*lacZ* fusion carried by pMLcsrBlac were determined in the wild type (HX2; circles) and a *barA* mutant (MR57; squares). (B) The relative level of ncRNA CsrB in *Rahnella aquatilis* MR57 with or without pRKbarA grown for 12 h in PDB medium. The amount of mRNA was determined by qPCR. Three replicates were used in this experiment. Data with the different letters are significantly different (*P*<0.05).

The global regulator BarA, a sensor protein of a two-component regulatory system BarA/UvrY, had been found as a regulation gene functioning in bacterial biocontrol effects in *R*. *aquatilis* HX2 (Table 2) [25]. BarA regulateed small noncoding regulatory RNAs in many other bacteria including *E*. *coli* and *P*. *fluorescens*. To determine the regulatory of CsrB by BarA in *R*. *aquatilis* HX2, a transcriptional fusion was conducted by placing the *lacZ* gene precisely at the +1 site under the control of the *csrB* promoter in a vector that has two to five copies per cell [38]. The activity of the *csrB* promoter increased with cell density and was 11–15 times higher at the late stationary phase in wild-type strain HX2 than BarA mutant (Fig 4A), which confirmed that the BarA/UvrY system is strongly involved in *csrB* transcription. Meanwhile, effect of BarA on the transcription of *csrB* was further examined by qPCR. Results showed that, compared with the wild type strain HX2 in PDB medium (normalized to 1), a significantly (*p*<0.01) lower level of *csrB* was observed in the BarA mutant strain MR57 without pRKbarA but no significant difference was observed in strain MR57 within pRKbarA (Fig 4B). In addition, the antagonism assay results showed that both BarA and CsrB restored the inhibition activity of BarA mutant MR57 *in vitro* (Table 2). However, BarA failed to restore the inhibition activity of the CsrB mutant MR61 (Table 2). These results suggested that CsrB is a part of the BarA/UvrY regulation of strain HX2 and required for the biocontrol regulation by BarA/UvrY regulation.

## Discussion

*R*. *aquatilis* HX2 is a biocontrol agent, and showed to inhibit the growth of phytopathogenic bateria including *A*. *tumefaciens*, *A*. *rhizogenes*, *A*. *vitis*, *Pectobacterium carotovorum*, *Xanthomonas campestris*, *X*. *oryzae*, *Pseudomonas syringae*, and *Clavibacter michiganensis* [19, 20]. Biocontrol activity of strain HX2 has been investigated for *A*. *vitis* in sunflowers and grapes in the greenhouse [20]. Moreover, biocontrol activity has been found to directly relate to the production of the antibacterial substance (ABS). In this study, a ncRNA (designated CsrB) was identified that transcription of this ncRNA is activated by the BarA/UvrY two-component system and positively regulated the production of ABS with the biocontrol activity. The predicted product of *csrB* was detected as an approximately 366-nt single transcript. Mutational inactivation of CsrB led to less production of ABS, leading to reduce biocontrol ability and similar properties of BarA mutant. The *csrB* promoter was also positively regulated by the BarA/UvrY system.

The ncRNAs act as the regulators of various cellular processes in bacteria [3]. These regulatory elements, also termed ribo-regulators, were categorized into several classes [39]. One class comprised the riboregulators that are able to establish base pairing with target mRNAs (e.g., DsrA, RprA, Spot42, MicF, UptR and RhyB) in *E*. *coli* [5, 40–42]. Such RNAs modified the behavior of the target mRNA upon binding under the assistance of the RNA-binding protein Hfq [39, 43]. A second important group of riboregulators, including CsrB and CsrC in *E*. *coli* [44, 45], CsrB in *S*. *typhimurium* [10], RsmB in *Erwinia* spp. [14] and RsmZ (PrrB) in *P*. *fluorescens* [46, 47], were antagonists of small translational regulators (CsrA, RsmA). The CsrB and CsrC RNAs of *E*. *coli* modulated the activity of CsrA, an RNA-binding protein that regulated carbon usage upon entry into stationary phase and other nutrient-poor conditions [48]. CsrA dimers binded to GGA motifs in the 5’-UTR of target mRNAs, thereby affected the stability and/or translation of the mRNA [48]. Each CsrB and CsrC RNAs contained multiple GGA binding sites. Thus, the ncRNAs effectively kept the CsrA protein away from mRNA leaders. Transcription of the *csrB* and *csrC* genes was induced by the BarA/UvrB two-component regulators when cells encountered nutrient-poor growth conditions though the signal for this induction is unknown.

**Fig 5:**
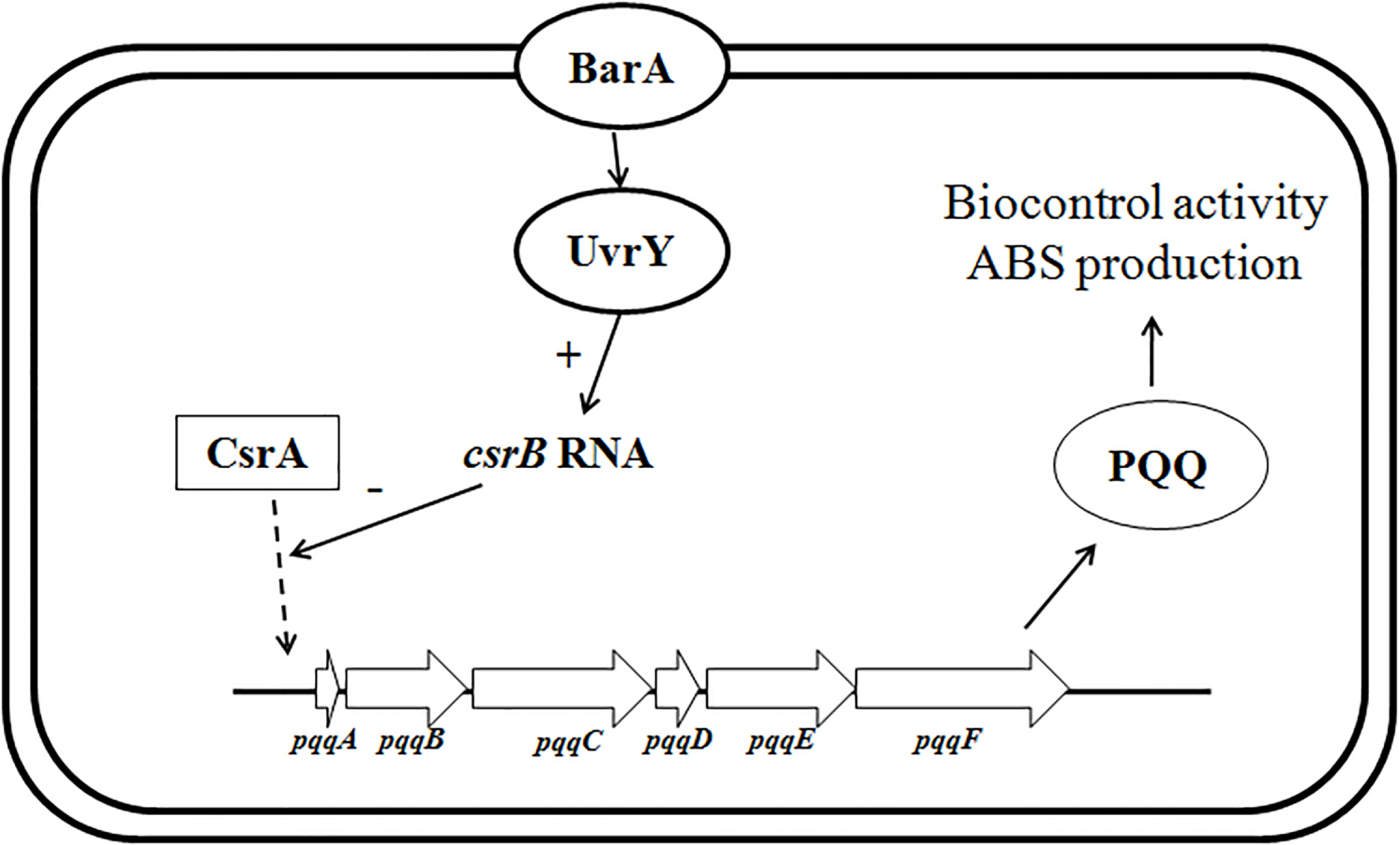
Model of regulation of *csrB*, BarA/UvrY and PQQ in the pathway of biocontrol activity and in ABS production of *R*. *aquatilis* HX2. The BarA/UvrY and PQQ regulatory cascades of *R*. *aquatilis* HX2 were adopted as described [23–25].

Our previous results showed that ABS production and biocontrol activity in *R*. *aquatilis* HX2, which were regulated by PQQ and the BarA/UvrY two component regulatory system [23–25]. In the current study, a novel ncRNA CsrB in *R*. *aquatilis* HX2 was further confirmed to play regulatory role in the ABS production and biocontrol activity against *A*. *vitis* on sunflower plants (Fig 5). Future research will be aimed at deciphering the network referring to ABS production and biocontrol activity as well as the genes regulated by the ncRNA CsrB.

## Supporting information

S1 Table DNA primers used in this study. (DOC)
