# Supplementary material for: CsrB, a noncoding regulatory RNA, is required for BarA-dependent expression of biocontrol traits in Rahnella aquatilis HX2
Source: PLoS One. 2017 Nov 1;12(11):e0187492. doi: 10.1371/journal.pone.0187492 (PMC5665550; doi:10.1371/journal.pone.0187492)
Supplement: S1 Table — (DOC) [file pone.0187492.s001.doc]

DNA primers used in this study

| **Primers** | Sequences (5’ to 3’)^a^ | Sources or references |
| --- | --- | --- |
| csrBKO1 | ACAGCGGCCGCACAGCGTCCAGATATCTGC | This study |
| csrBKO2 | GAGGAATGGCTCTTCATGCTTCCTTAGTGC | This study |
| csrBKO3 | GAAGCATGAAGAGCCATTCCTCAGCCGATC | This study |
| csrBKO4 | ACAGCGGCCGCTCACCATTCTGATGTTCTC | This study |
| csrBCO1 | ACAAAGCTTCGACACAGTATGCAGGG | This study |
| csrBCO2 | ACAGGATCCCTGGTGATCGGCATGCTG | This study |
| csrBPR1 | ACAGGATCCTCTTCTGGCTAACACAG | This study |
| csrBPR2 | ACACTGCAGTAGAATCGTTGATTTCG | This study |
| csrBqRT1 | GGACGGCTCAGGAAGAG | This study |
| csrBqRT2 | ACGGTCTGTCCTAAGTC | This study |
| csrBRA1 | TTCATCCTGACGGTCTG | This study |
| DT88 | GAAGAGAAGGTGGAAATGGCGTTTTGG | [30] |
| DT89 | CCAAAACGCCATTTCCACCTTCTCTTC | [30] |
| rplUFor | TCTGGAGAAGCTGGACGT | This study |
| rplURev | CTGCTGCTTACGATAGTG | This study |
